# Health Care Provider Perceptions of Screening for Early‐Stage Type 1 Diabetes—A Survey Study

**DOI:** 10.1111/dom.70803

**Published:** 2026-04-22

**Authors:** Claire Cherdron, Peter Achenbach, Kathrin Ackermann, Florian Haupt, Anja Heublein, Annette Knopff, Sarah Schill, Jennifer Schmidt, Joanna Stock, Christiane Winkler, Anette‐G. Ziegler, Sandra Hummel

**Affiliations:** ^1^ Institute for Medical Information Processing Biometry, and Epidemiology—IBE, LMU Munich Munich Germany; ^2^ Pettenkofer School of Public Health Munich Germany; ^3^ Institute of Diabetes Research, Helmholtz Munich, German Research Centre for Environmental Health Neuherberg Germany; ^4^ Technical University of Munich, School of Medicine and Health, Forschergruppe Diabetes, TUM University Hospital Munich Germany; ^5^ Forschergruppe Diabetes e.V. at Helmholtz Zentrum München Munich Germany; ^6^ German Centre for Diabetes Research (DZD) Munich Germany

**Keywords:** autoimmunity, population study, primary care, type 1 diabetes

## Abstract

**Aims:**

To evaluate the feasibility, perceived benefits, and challenges of integrating early‐stage Type 1 diabetes screening and monitoring into routine care.

**Materials and Methods:**

Primary health care providers (HCPs; *n* = 683), responsible for collecting capillary blood samples and obtaining informed consent, and specialised diabetes centres (*n* = 17), responsible for monitoring children with early‐stage Type 1 diabetes using oral glucose tolerance tests (OGTT) and HbA1c, within an early‐stage Type 1 diabetes screening programme in Bavaria, Germany (Fr1da) were invited to participate in an online survey.

**Results:**

Among the 194 responding primary HCPs, 66% rated overall feasibility of integrating screening in routine care as ‘very good’ or ‘good’. More than 70% rated informing families about screening and communicating results positively, while 54% rated capillary blood sampling positively. Among the 10 responding diabetes centres, ≥ 80% rated feasibility of OGTT and HbA1c positively. Families' acceptance of glucose (on‐site or at‐home) and HbA1c monitoring was perceived as high. Screening was considered beneficial by 91% of primary HCPs and by all diabetes centres, emphasising reduced efforts for insulin initiation and long‐term care. Reported key challenges included time and staffing constraints and inadequate reimbursement.

**Conclusions:**

Routine‐care implementation of early‐stage Type 1 diabetes screening is broadly supported by primary HCPs and diabetes centres and could be facilitated by refined workflows and appropriate reimbursement.

**Abbreviations**
HCP: Health care provider
IQR: Interquartile Range
OGTT: Oral glucose tolerance test

## 1 Introduction

Type 1 diabetes follows a progressive course, with early‐stage Type 1 diabetes defined by the presence of two or more islet autoantibodies preceding the clinical onset of the disease [1]. Diagnosing early‐stage Type 1 diabetes substantially reduces the risk of severe metabolic decompensation at clinical onset [2, 3, 4] and enables disease‐modifying therapies that delay disease progression [5], highlighting the increasing importance of identifying children in early stages for preventive care.

Public health screening for early‐stage Type 1 diabetes and monitoring of children in early stages has proven feasible in research settings [6, 7], and legislation supporting such screening has been introduced in Europe [8]. Projections suggest that nationwide implementation could temporarily increase the number of children requiring specialised care by up to 60% [9], emphasising the need for efficient and sustainable implementation strategies. However, knowledge about health care providers' (HCP) perception of such screening programmes remains limited.

We conducted a survey among primary HCPs and specialised paediatric diabetes centres participating in an ongoing screening programme for early‐stage Type 1 diabetes to assess their perceptions of the feasibility, benefits, and challenges of integrating early‐stage Type 1 diabetes screening into routine paediatric care.

## 2 Methods

### 2.1 Survey Design

Investigator‐designed, web‐based surveys were conducted among primary HCPs and specialised paediatric diabetes centres to evaluate their perceptions of the feasibility, benefits, and challenges of integrating early‐stage Type 1 diabetes screening of children within routine workflows. The surveys assessed respondents' experiences within a research setting as well as their perceptions of implementing screening and monitoring beyond the study context. The survey for primary HCPs included 33 items, and the survey for diabetes centres comprised 37 items (Methods S1) [6]. Quantitative items employed a five‐point Likert scale to capture respondents' levels of agreement or satisfaction, ranging from *strongly disagree/very poor* (1) to *strongly agree/very good* (5). Additional questions assessed the time required for individual screening and monitoring procedures and explored diabetes centres' perceptions of monitoring procedures. Open‐ended questions were included for qualitative data collection.

Primary HCPs and diabetes centres participating in an ongoing public health screening programme for early‐stage Type 1 diabetes in children aged 1.75 to 10.99 years (Fr1da‐study) were invited by email to participate in the surveys. The survey was open from March 29 to May 31, 2025. Participation was voluntary and anonymous, and reimbursement of 50€ per completed questionnaire was offered.

### 2.2 Workflow of Screening for Early‐Stage Type 1 Diabetes

Screening for early‐stage Type 1 diabetes is accomplished by primary HCPs in collaboration with a coordinating centre (Figure S1). Screening is recommended during routine well‐child visits, with the first screening at age 3 years and, if the results are negative, a rescreening at age 7 years. The workflow for primary HCPs consists of recruiting families, obtaining informed consent to participate in the Fr1da study, and collecting capillary blood (200 μL) and demographic data. Samples are stored refrigerated and returned regularly to the central laboratory for islet autoantibody testing. If two or more islet autoantibodies test positive, primary HCPs are asked to obtain a venous blood sample for confirmatory testing. Upon confirmation by the central laboratory, primary HCPs are responsible for communicating the diagnosis of early‐stage Type 1 diabetes to families. Families are referred to a diabetes centre near their home for metabolic staging, monitoring, and educational training.

### 2.3 Workflow of Monitoring Children With Early‐Stage Type 1 Diabetes

Monitoring of children diagnosed with early‐stage Type 1 diabetes is accomplished by specialised paediatric diabetes centres. Their workflow consists of metabolic staging by using an oral glucose tolerance test (OGTT) and HbA1c measurement. Tailored to the results of the metabolic staging, an individualised schedule for future monitoring, including blood glucose, HbA1c and/or OGTT, is implemented by the coordinating centre (Figure S1). Families are additionally offered educational training on early‐stage Type 1 diabetes.

### 2.4 Data Analysis

Descriptive statistics were used for participant characteristics, with results reported as absolute numbers (*n*) and percentages (%) or median and interquartile range (IQR), and to calculate the distribution (%) of responses for quantitative survey items across the five‐point Likert scale. Estimated cumulative time for screening and monitoring was calculated based on reported times for individual procedures outlined in Figure S1. Subgroup comparisons were performed using the Wilcoxon rank sum test. A two‐sided *p* value of < 0.05 was considered statistically significant. Quantitative data analysis was performed using R version 4.3.1.

A qualitative content analysis was conducted using an inductive‐deductive approach as described by Mayring [10]. A coding manual was initially developed by the first author (C.C.), drawing on constructs from the Consolidated Framework for Implementation Research [11], and reviewed by S.H. To assess coding reliability, a random subset of responses was independently coded by a second researcher (K.A.). Only minor discrepancies were observed, which were resolved through discussion.

Quantitative and qualitative data were analysed independently and subsequently integrated to combine numerical trends with contextual insights, enabling a comprehensive assessment of feasibility, perceived benefits, and challenges of integrating early‐stage Type 1 diabetes screening into routine workflow.

## 3 Results

### 3.1 Primary HCPs

Of the 683 invited primary HCPs, 194 (28%) completed the online survey, including 82% physicians (Table 1). Among all respondents, the median number of children screened per month was five (IQR 1–12), 126 (65%) were participating in the screening programme for more than 5 years, and 118 (61%) offered a rescreening. Among those, the median proportion of children who were rescreened was 10% (IQR 2%–20%). In addition, 124 of 194 (64%) primary HCPs indicated that at least one child had been diagnosed with early‐stage type 1 diabetes through the screening programme. The median proportion of families declining screening was 40% (IQR 10%–70%, Table 1) among primary HCPs who provided this information (*n* = 117).

**TABLE 1:** Characteristics of primary healthcare providers and specialised paediatric diabetes centres responding to the online survey.

	Number of respondents (*N*)	*N* (%) or median (IQR)
Primary healthcare providers		
Professional role of survey respondent, *n* (%)	194	
Physician		160 (82)
Nurse		34 (18)
Duration of participation in the screening programme, *n* (%)	194	
< 1 year		8 (4)
1–5 years		60 (31)
> 5 years		126 (65)
Number of children screened per month, Median (IQR)	194	5 (1, 12)
Percentage of families declining screening, Median (IQR)	117	40 (10, 70)
Offering rescreening for children with a negative screening result, *n* (%)	194	
Yes		118 (61)
No		41 (21)
Not aware of rescreening		35 (18)
Percentage of children offered rescreening, Median (IQR)	111	10 (2, 20)
Experience with the diagnosis of early‐stage T1D within the screening programme, *n* (%)	194	
Yes		124 (64)
No		70 (36)
Offering monitoring for children with early‐stage Type 1 diabetes, *n* (%)	181	
Yes		78 (43)
No		103 (57)
Specialised paediatric diabetes centres		
Professional role of survey respondent, *n* (%)	10	
Physician		7 (70)
Nurse		1 (10)
Other		2 (20)
Duration of participation in the screening programme, *n* (%)	10	
1 year		0 (0)
1–5 years		2 (20)
> 5 years		8 (80)
Number of children diagnosed with early‐stage Type 1 diabetes through screening currently under care, Median (IQR)	10	10 (6, 20)

Abbreviation: IQR: Interquartile range.

Of the 194 primary HCPs, 81 (42%) also provided responses to open‐ended questions on challenges, and 33 (17%) reported additional suggestions, benefits, or concerns related to implementing screening into routine care outside a research setting (Methods S1).

### 3.2 Feasibility of Screening for Early‐Stage Type 1 Diabetes

**FIGURE 1:**
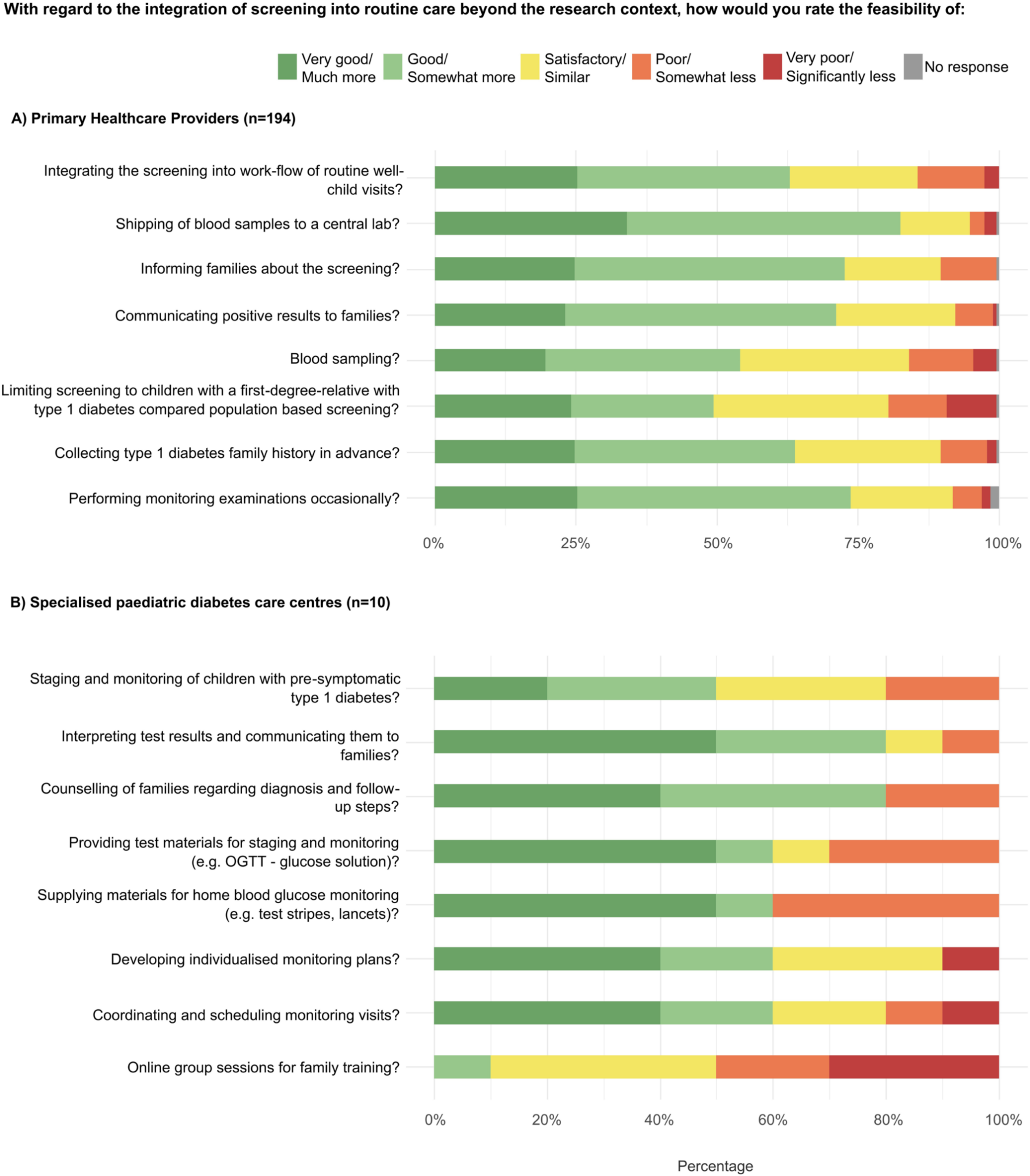
Feasibility of integrating public health screening for early‐stage Type 1 diabetes, as reported by primary healthcare providers (A), and monitoring of children with early‐stage Type 1 diabetes, as reported by specialised diabetes centres (B) into routine care beyond the research context. Results show feasibility overall and for individual screening and monitoring procedures.

Based on experience with screening implementation in a research setting, 66% of responding primary HCPs considered the feasibility of screening for early‐stage Type 1 diabetes within routine workflows as ‘very good’ or ‘good’, and 63% rated its feasibility within well‐child visits positively. Similarly, 63% considered its integration into routine well‐child visits feasible beyond the research setting (Figure 1A; Figure S2).

Individual screening‐related procedures, including informing families, sample shipment, and communication of positive results, were also predominantly rated positively for implementation into routine care outside the research context (Figure 1A). Capillary blood sampling was rated positively by 54% of respondents, while 16% rated it as ‘rather poor’ or ‘very poor’. Targeted screening of children with a first‐degree family history of Type 1 diabetes was perceived as more feasible than population‐based screening by 50% of respondents, and 64% considered assessments of family history feasible (Figure 1A). Among the 181 primary HCPs who answered the respective question, 78 (43%) reported having already performed HbA_1c_ or OGTT for staging or monitoring children with early‐stage Type 1 diabetes (Table 1). Regardless of prior experience, 75% of the 194 respondents rated occasional performance of such examinations positively (Figure 1A). Open‐ended responses further supported the feasibility of implementing screening outside the research setting and suggested potential benefits for workflow standardisation and parental acceptance (Table 2).

**TABLE 2:** Categories, subcategories, and representative quotes illustrating challenges, perceived benefits, and improvement suggestions of integrating screening for early‐stage Type 1 diabetes into routine care beyond the research context, reported by primary healthcare providers and specialised paediatric diabetes centres.

Category	Sub‐categories	Representative quote primary healthcare providers	Representative quote specialised paediatric diabetes centres
1. Process‐related challenges	1.1 Issues with study workflow	‘Delay in consultation time due to study participation during/after routine check‐ups.’ (604) ‘The blood volume required for capillary blood collection is relatively high, which often leads to unsuccessful collection on the first attempt (because the volume cannot be collected completely). In such cases, either a repeat capillary blood sample is required, or venous blood sampling is performed.’ (483)	‘Development of the individual monitoring plan, as there is currently no standardised procedure’ (805) ‘We observe children with early insulin insufficiency, identifying the appropriate time to start insulin therapy remains difficult.’ (780)
	1.2 Age limitations	‘Sometimes the age limits are annoying.’ (369)	—
2. Resource‐related challenges	2.1 Issues with study materials	‘The capillary is very thick resulting in poor blood flow.’ (340)	‘Ensuring that all necessary materials are available at all times is challenging.’ (810)
	2.2 Time constraints	‘It's just very time‐consuming—you must explain that they have the option for screening and that a blood sample is required. And why [the screening] is useful.’ (541)	‘Staffing shortages, as a large number of children with diabetes are already being cared for, which entails considerable challenges and effort. In particular, establishing and coordinating follow‐up care is very time‐consuming.’ (783)
	2.3 Staff shortages	‘However, blood sampling ties up a nurse who is then missing elsewhere.’ (587)	‘Integration requires a lot of specialised medical and psychological personnel who need to be available also on weekends.’ (811)
	2.4 Inadequate compensation (practice level)	‘The reimbursement of €10 per child is not covering the effort involved.’ (541)	—
3. System‐level challenges	3.1 Inadequate system‐level reimbursement	‘In routine care, the overall effort required is likely to be reimbursed even less adequately. Health insurance providers may again attempt to include this procedure under existing well‐child visit billing codes without providing additional compensation.’ (433)	‘Reimbursement by the health insurance providers must be ensured.’ (780)
	3.2 Insufficient infrastructure of specialised paediatric diabetes centres	‘The centres to which children are referred following positive screenings are often far from the families' homes, requiring parents to travel long distances. Expanding the number of referral centres would therefore be highly desirable.’ (530)	‘Currently, however, all paediatric diabetes centres are operating at full capacity. Without well‐distributed follow‐up care, integrating screening into standard healthcare is likely to be very challenging.’ (811)
4. Individual‐level: Parental and child‐related challenges	4.1 Acceptability and perceived disadvantages	‘Many parents are reluctant to blood draws’ (428) ‘Children often resist, experience fear; worried that the negative experience will leave a lasting impression.’ (604)	‘Willingness of all parents to participate’ (805)
	4.2 Parental right not to know	‘It is also questionable whether families really want to know whether this serious illness will 1 day affect their family.’ (417)	—
	4.3 Parental scepticism toward medicine and research	‘Overall, there are more and more parents who refuse vaccinations and other things, or mention that the wish to discuss participation with the midwife or osteopath first.’ (467)	—
	4.4 Lack of health literacy	‘Lack of health literacy in the general population’ (602)	—
	4.5 Language challenges	‘Language barrier for foreign patients’ (364)	—
5. Individual‐level: Healthcare professionals' attitudes and acceptance	5.1 Negative perception on invasiveness, usefulness and disadvantages	‘Personally, I would decide against [the screening], but I still offer it.’ (417) ‘The rather limited benefit of pre‐symptomatic diagnosis.’ (577)	‘More important than screening is clear education on the most common symptoms of diabetes in the yellow child health booklet (U‐Heft), as these symptoms are still frequently misdiagnosed as urinary tract or gastrointestinal infections, sometimes even by general practitioners and paediatricians.’ (813)
	5.2 Lack of Motivation	‘Not all primary care paediatricians are equally committed’ (414)	—
	5.3 Positive attitudes	‘The study is very well designed and feasible’ (542) ‘I don't see any major challenges in integrating screening into standard care.’ (389)	‘Screening is useful! Participation is perceived as meaningful and valuable. The (additional) work is perceived as rewarding.’ (780)
6. Suggested Improvements		‘Acceptance of the prick [is challenging], would increase with the filter card, as the parents know this from the newborn screening.’ (390) ‘Routine information for parents before screening would be helpful ‘(344) ‘An idea for the future would be saving paper and questionnaires: perhaps eventually there will be the possibility to record the consent form and medical history digitally via tablet and transmitting them electronically?’ (378)	‘Staging should be standardised with a concrete plan/flow chart.’ (780)
7. Anticipated benefits		‘If it became a standard service, we would develop a fixed routine for its implementation. […] I believe that if the screening was offered as a routine service, acceptance would increase significantly, as it would allow us to provide information in a different way. Furthermore, an additional appointment would be avoided, which would be good.’ (587) ‘Parents who themselves have type 1 diabetes are always immediately motivated; there is no difficulty in conveying the value of screening to them. In my view, however, implementing testing would be particularly beneficial for all other families as well.’ (527)	‘All Fr1da specialised diabetes centres will likely have no issues with the medical materials, as these are routinely kept in stock. This will probably even simplify matters, as the teams are more familiar with their own materials.’ (789)

*Note*: The participant identification number is indicated in parentheses after each quotation.

### 3.3 Benefits of Screening for Early‐Stage Type 1 Diabetes

The majority of responding primary HCPs rated the screening as clinically useful (91%) and beneficial for families (77%, Figure S2), highlighting the importance of screening in families without a prior history of the disease (Table 2), while four respondents emphasised parents' right not to know about a potential disease (Table 2). Additionally, 76% of responding HCPs reported gaining new knowledge about Type 1 diabetes through their participation in the screening programme (Figure S2).

### 3.4 Challenges of Screening for Early‐Stage Type 1 Diabetes

Qualitative data analysis identified five major categories of challenges (Table 2). Among them, *resource‐related challenges* were mentioned most frequently, including time and staff constraints. The informed consent process was reported as particularly time‐consuming, especially when families were unfamiliar with the screening programme or had limited knowledge of Type 1 diabetes. The perceived workload for screening was rated as ‘rather high’ or ‘very high’ by 21% of respondents, while 39% rated it as ‘very low’ or ‘low’ (Figure S2). Primary HCPs reported a median cumulative time of 17 min (IQR 13–22) to complete all steps required for screening when the child tested negative, and of 49 min (IQR 38–59) for children diagnosed with early‐stage Type 1 diabetes (Table S1). Primary HCPs who reported a ‘very low’ or ‘low’ workload reported significantly shorter time required for screening than those who reported average or higher workload. This applied to both children with negative screening results (median 15 min [IQR: 12–19] vs. 19 min [IQR: 14–25], *p* = 0.002) and children diagnosed with early‐stage Type 1 diabetes (median 43 min [IQR: 35–55] vs. 51 min [IQR: 42–61], *p* = 0.013). Primary HCPs who screened more than 10 children per month required less cumulative time for screening than those screening fewer children (*p* < 0.001 for children with a negative screening result; *p* = 0.003 for children diagnosed with early‐stage Type 1 diabetes, Table S1).

Respondents also noted *process‐related challenges*, including workflow issues such as insufficient time for families to consider participation during well‐child visits, difficulties with required capillary blood volume, and concerns that age eligibility criteria did not align with routine well‐child visit schedules.

*System‐level challenges* included concerns about inadequate reimbursement for screening and limited availability and capacity of specialised care centres for monitoring children with early‐stage Type 1 diabetes, particularly in rural regions.

*Parental and child‐related challenges* included families' willingness to participate, children's non‐compliance with blood sampling, skepticism toward conventional medicine, language barriers, and limited health literacy. Responding primary HCPs emphasized the importance of voluntary participation.

Primary HCPs' responses also emphasised the importance *of healthcare professionals' attitudes and acceptance* of screening, including their concerns about invasiveness, perceived usefulness of the screening, and individual motivation.

Additionally, several primary HCPs suggested improvements to enhance implementation, including digitalisation of data collection, process optimization (e.g., reducing required blood volume or using dried blood spot cards), and expanded public education. Respondents also recommended collecting screening samples during clinically indicated venous blood draws to improve efficiency.

### 3.5 Specialised Paediatric Diabetes Centres

Among the 17 invited diabetes centres, 10 (59%) completed the survey and responded to at least one of the open‐ended questions on challenges, suggestions for improvement, perceived benefits, or concerns related to implementing screening and monitoring into routine care outside a research setting. Most respondents were physicians (*n* = 7), and eight of 10 centres had participated in the Fr1da study for more than 5 years. Among all responding centres, a median of 10 children (IQR 6–20) were currently under monitoring (Table 1).

### 3.6 Feasibility of Monitoring Children With Early‐Stage Type 1 Diabetes

All responding diabetes centres rated staging and monitoring in collaboration with a coordinating centre as ‘very good’ or ‘good’, based on their experience within the research context (Figure S3). For implementation beyond the research setting, 50% of responding centres considered integrating staging and monitoring into routine care as ‘very good’ or ‘good’. Several centres emphasised the importance of coordinated support, such as the provision of monitoring supplies, to maintain a manageable workload (Figure 1B, Table 2).

**FIGURE 2:**
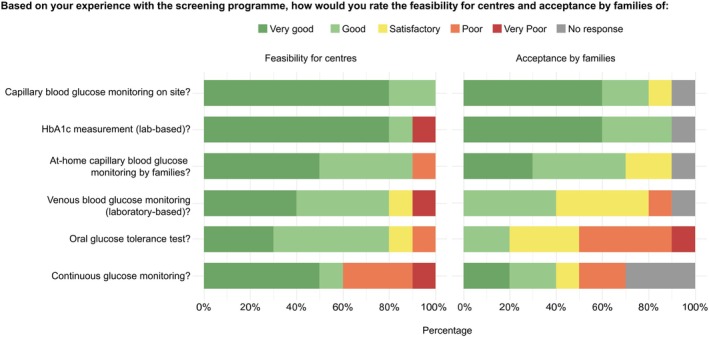
Feasibility and family acceptance of individual procedures related to monitoring children with early‐stage Type 1 diabetes, as reported by specialised paediatric diabetes centres based on their experience with the screening programme for early‐stage Type 1 diabetes.

Among monitoring procedures, capillary blood glucose measurement was considered the most feasible and best accepted followed by laboratory‐based HbA_1c_ measurement, particularly when using capillary sampling and point‐of‐care testing (Figure 2). At‐home capillary blood glucose monitoring was rated positively by 80% of centres, and 70% noted good family acceptance following initial training. Venous blood glucose measurements and OGTTs were each rated positively by 80% of centres; however, 90% identified OGTTs as among the most challenging monitoring procedures due to time requirements, child distress, and lower family acceptance, particularly among those travelling long distances (Figure 2, Table 2). As potentially more feasible alternatives to the standard OGTT, five centres suggested capillary or CGM‐based OGTTs, two proposed reducing the number of OGTT timepoints, and one suggested capillary‐based HbA_1c_ or blood glucose measurements by local primary HCPs. While 50% of the centres rated the feasibility of CGMs as ‘very good’, 40% rated it as ‘poor’ or ‘very poor’ due to limited sensor availability, unsecure health insurance coverage, and technical demands (Figure 2). Nevertheless, identifying optimal timing for insulin therapy initiation was cited as an advantage of CGM.

### 3.7 Benefits of Monitoring Children With Early‐Stage Type 1 Diabetes

**FIGURE 3:**
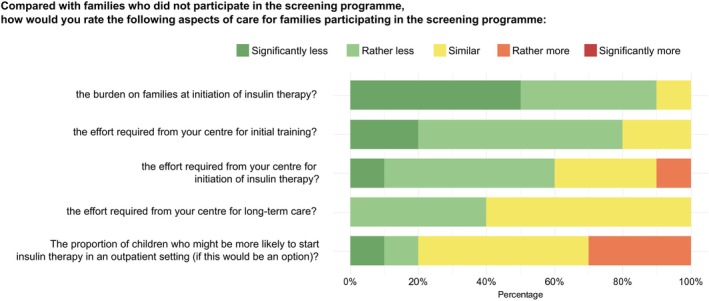
Benefits of early‐stage Type 1 diabetes screening and monitoring, as reported by specialised paediatric diabetes centres.

Most responding specialised care centres (90%) viewed screening for and monitoring of children with early‐stage Type 1 diabetes as clinically useful (Figure S3). Reported advantages included a lower burden at insulin initiation, as well as less effort for initial training and insulin initiation, attributed to established relationships with families and psychological preparation. Some centres also noted a reduced effort for long‐term care (Figure 3).

Furthermore, 30% of centres estimated that children identified in early stages might be more likely to start insulin therapy in an outpatient setting, if this became an option (Figure 3). Respondents further emphasised the reduced risk of DKA at clinical onset and the growing relevance of public health screening given emerging therapeutic options, while one centre prioritised public awareness campaigns over screening for early‐stage Type 1 diabetes (Table 2). Additionally, 80% of centres reported gaining new knowledge about Type 1 diabetes through their participation in the screening programme, and 30% indicated a continued need for training (Figure S3).

### 3.8 Challenges of Monitoring Children With Early‐Stage Type 1 Diabetes

Qualitative data analysis identified that challenges reported by diabetes centres (*n* = 10) closely reflected those reported by primary HCPs (Table 2) and were largely *process‐* and *resource‐*related. These included the need to plan monitoring visits individually with families due to staging‐dependent follow‐up schedules and limited capacity to manage additional workload because of staff availability (Table 2). Overall, the reported time required for staging or monitoring visits was not associated with the number of children under care (Table S1).

Additionally, one centre noticed uncertainty regarding the timing of insulin initiation, underscoring the importance of support by a coordinating institution. Offering online group family training to reduce workload was considered less useful compared with individualised training by 50% of the centres (Figure 1B). The need for policy frameworks to ensure coverage by health insurance providers appeared as the most cited *System‐level challenge*, alongside concerns about sufficient availability of specialised care centres to monitor children with early‐stage Type 1 diabetes. Additionally, *parental and patient‐related challenges* included parental willingness to participate with their child and emotional stress associated with uncertainty about disease progression. Small incentives for children were reported to improve the overall experience.

## 4 Discussion

This survey study demonstrates that most primary HCPs and diabetes centres perceive feasibility of screening for early‐stage Type 1 diabetes as ‘very good’ or ‘good’ and as clinically beneficial, based on their experience within a research setting. The analysis further identified challenges to integrating screening and monitoring into routine workflows beyond the research context and highlights potential strategies to facilitate implementation.

The supportive attitudes of most primary HCPs and diabetes centres toward early‐stage Type 1 diabetes screening align with findings from Italy, where 84% of primary HCPs expressed willingness to participate in a national screening programme [12], and from the U.S., where 52% of endocrinology HCPs supported screening following FDA approval of teplizumab [13]. Notably, all respondents in our study had prior experience of integrating screening into routine workflow through research participation. In contrast, only 26% of Italian respondents understood their role in the screening process, highlighting the importance of structured implementation frameworks and ongoing provider education [14].

Among individual screening procedures, most primary HCPs rated informing families about the screening, communicating test results, and shipping blood samples to a central laboratory positively. Capillary blood collection was rated positively by 54% of respondents, although some primary HCPs found it challenging, mainly due to the required sample volume of 200 μL. Some respondents suggested the use of dried blood spots, like those employed in newborn screening. While dried blood spots have been explored for islet autoantibody screening [15, 16, 17], their applicability remains limited, as current assay technologies still require larger blood volumes than those typically used in newborn screening. In addition, occasional monitoring visits in primary care were considered feasible by three‐quarters of primary HCPs, which could help ensure monitoring capacity, particularly in rural areas.

Likewise, most diabetes centres rated the feasibility of individual monitoring procedures positively and reported high acceptance by families for capillary blood glucose and HbA1c. While 80% of diabetes centres rated the feasibility of performing OGTTs positively, perceived family acceptance of OGTTs was the lowest, leading to suggestions for less‐invasive alternatives. Among these, HbA_1c_ provides high specificity for diagnosing stage 3 Type 1 diabetes and may be comparable to OGTT in predicting disease progression when measured longitudinally [14, 18]. Its suitability for capillary sampling and point‐of‐care testing further supports implementation into routine care [14, 19], alongside its high perceived acceptance by families.

Based on findings from this survey, participation in screening for early‐stage Type 1 diabetes appears to enhance disease‐related knowledge among HCPs, which may contribute to improvements in the overall quality of care for affected children. In line with this, most respondents rated screening for early‐stage Type 1 diabetes as clinically beneficial, consistent with findings form surveys conducted in the United States [13] and Italy [12]. In addition, several diabetes centres reported that early‐stage diagnosis was associated with lower requirements for initial training and long‐term care, and 30% anticipated that early diagnosis would reduce the number of children requiring hospitalisation for insulin initiation. These effects could contribute to reduced workload and healthcare costs compared with later‐stage diagnosis. This is important, as some respondents perceived limited time as a major challenge to implementing screening programmes in routine care. While most primary HCPs rated the feasibility of informing families about the screening and obtaining consent positively, some reported it to be time‐consuming, highlighting the value of providing informational materials ahead of appointments and strengthening public awareness of screening. Moreover, screening efficiency appeared to improve with experience, implying that structured learning from screening‐experienced primary HCPs may help reduce resource demands. In this context, several diabetes centres expressed a need for support from a coordinating structure, at least for transition into routine care beyond the research setting, such as provision of supplies or scheduling follow‐up visits with families according to the individual monitoring plan. Digital infrastructure, including AI‐based approaches, may assist with personalised monitoring [20].

Finally, some respondents reported concerns about inadequate reimbursement for screening and monitoring, underscoring the need for supportive policy frameworks and sustainable reimbursement models. Recently published cost estimates for public health screening and monitoring may provide a basis for developing such reimbursement strategies [21]. In addition, the introduction of the ICD‐10 diagnosis codes E10.A1 and E10.A2 in the United States, as well as R76.80 and R73.00 within the WHO ICD‐10 classification used in Germany, for early‐stage Type 1 diabetes represents an important step toward laying the foundation for healthcare coverage and reimbursement within routine care.

Many challenges identified in our study mirror those reported from other clinical implementation efforts [12, 13, 22, 23], although cross‐country comparisons may be limited by differences in healthcare systems, screening strategies, and policy environments.

This study is limited by a moderate response rate and its regional focus on Bavaria, where the screening programme was first established; therefore, the findings may not be fully generalisable to other regions. Although the absolute number of specialised paediatric diabetes centres involved in the screening programme is small, the sample represents a substantial proportion of centres within the region. The findings may also be subject to selection bias, as more engaged HCPs were likely more inclined to participate in both the screening programme and the survey, potentially resulting in more favourable assessments of feasibility. In addition, the structured survey format may have limited the depth of qualitative insights, and despite framework‐guided coding, generalisability of qualitative responses remains limited. Given the complexity of implementing screening beyond the research setting, caution is warranted when extrapolating these findings to routine care, particularly among HCPs without prior screening experience. Further research is needed to evaluate infrastructural and policy‐related factors and family perspectives. Strengths of this study include the anonymous survey design and real‐world insights from both primary HCPs and diabetes centres with experiences of integrating screening and monitoring into their routine workflow within a research context, enabling a more realistic assessment of feasibility and associated challenges compared to responses from healthcare professionals without any screening experience.

In conclusion, most primary HCPs and diabetes centres rated the feasibility of integrating early‐stage type 1 diabetes screening and monitoring into routine care positively and perceived it as clinically beneficial. Key benefits included lower rates of DKA, a reduced burden for families during insulin initiation, decreased effort required from diabetes centres during insulin initiation and long‐term care, enhanced HCP knowledge, and improved prospects for the uptake of emerging therapies.

Time and staffing constraints, along with inadequate reimbursement, were identified by some primary HCPs and diabetes centres as challenges to implementation beyond the research setting. Increasing public awareness, providing informational materials prior to screening, support from a coordinating institution to supply materials and coordinate individualised monitoring, as well as sustainable reimbursement frameworks, may facilitate integration into routine paediatric care.

## Author Contributions

Clarie Cherdron, Christiane Winkler, Anette‐G. Ziegler and Sandra Hummel were responsible for the design of the survey. Clarie Cherdron, Kathrin Ackermann and Sandra Hummel performed data analysis. Joanna Stock, Anja Heublein, Annette Knopff and Jennifer Schmidt researched the data. Anette‐G. Ziegler was responsible for the Fr1da study design. Peter Achenbach, Florian Haupt, Christiane Winkler and Anette‐G. Ziegler were responsible for Fr1da study conduct. Anette‐G. Ziegler is the principal investigator of the Fr1da study. Clarie Cherdron and Sandra Hummel drafted the manuscript. All authors reviewed and approved the final version of the manuscript. Sandra Hummel is the guarantors of this work and, as such, had full access to all the data in the study and takes responsibility for the integrity of the data and the accuracy of the data analysis.

## Funding

The survey was funded by Breakthrough T1D (# 3‐SRA‐2026‐1795‐S‐B; Funding for this grant is made possible through the collaboration between Breakthrough T1D and The Leona M. and Harry B. Helmsley Charitable Trust) and the EASD‐Novo Nordisk Foundation Diabetes Prize for Excellence (NNF22SA0081044). The funding sources were not involved in the design or conduct of the study; the collection, management, analysis, or interpretation of the data; the preparation, review, or approval of the manuscript; or the decision to submit the manuscript for publication.

## Conflicts of Interest

The authors declare no conflicts of interest.

## Supporting information


**Table S1:** Estimated time per child for early‐stage Type 1 diabetes screening and monitoring. Data are shown as total, reflecting responses of all responding primary healthcare providers respectively specialised paediatric diabetes centres, and stratified by the number of children screened respectively under care per month.
**Figure S1:**. Flow‐chart of the procedures and responsibilities within the early‐stage Type 1 diabetes screening programme.
**Figure S2:**. Feasibility of screening for early‐stage Type 1 diabetes within a research setting, as reported by primary health care providers.
**Figure S3:**. Feasibility of monitoring children with early‐stage Type 1 diabetes within a research setting, as reported by specialised paediatric diabetes centres.
**Methods S1**. Survey items for primary and specialised HCP.

## Data Availability

Data requests will be honoured from researchers who provide a methodologically sound proposal and who complete a Data Use Agreement with Helmholtz Munich. Requests should be directed by email to the corresponding author.

## References

[1] R. A. Insel , J. L. Dunne , M. A. Atkinson , et al., “Staging Presymptomatic Type 1 Diabetes: A Scientific Statement of JDRF, the Endocrine Society, and the American Diabetes Association,” Diabetes Care 38, no. 10 (2015): 1964–1974, 10.2337/dc15-1419. 26404926 PMC5321245

[2] S. Hummel , J. Carl , N. Friedl , et al., “Children Diagnosed With Presymptomatic Type 1 Diabetes Through Public Health Screening Have Milder Diabetes at Clinical Manifestation,” Diabetologia 66, no. 9 (2023): 1633–1642, 10.1007/s00125-023-05953-0. 37329450 PMC10390633

[3] C. Winkler , E. Schober , A. G. Ziegler , and R. W. Holl , “Markedly Reduced Rate of Diabetic Ketoacidosis at Onset of Type 1 Diabetes in Relatives Screened for Islet Autoantibodies,” Pediatric Diabetes 13, no. 4 (2012): 308–313, 10.1111/j.1399-5448.2011.00829.x. 22060727

[4] H. Elding Larsson , K. Vehik , R. Bell , et al., “Reduced Prevalence of Diabetic Ketoacidosis at Diagnosis of Type 1 Diabetes in Young Children Participating in Longitudinal Follow‐Up,” Diabetes Care 34, no. 11 (2011): 2347–2352, 10.2337/dc11-1026. 21972409 PMC3198296

[5] A. G. Ziegler , E. Cengiz , and T. W. H. Kay , “The Future of Type 1 Diabetes Therapy,” Lancet 406, no. 10511 (2025): 1520–1534, 10.1016/s0140-6736(25)01438-2. 40983070

[6] K. Kick , V. S. Hoffmann , K. Lange , et al., “Feasibility and Organization of a Population‐Based Screening for Pre‐Symptomatic Type 1 Diabetes in Children — Evaluation of the Fr1da Study,” Journal of Public Health 27, no. 5 (2018): 553–560, 10.1007/s10389-018-0981-x.

[7] P. D. Gesualdo , K. A. Bautista , K. C. Waugh , et al., “Feasibility of Screening for T1D and Celiac Disease in a Pediatric Clinic Setting,” Pediatric Diabetes 17, no. 6 (2016): 441–448, 10.1111/pedi.12301. 26251221 PMC4979315

[8] E. Bosi and C. Catassi , “Screening Type 1 Diabetes and Celiac Disease by Law,” Lancet Diabetes & Endocrinology 12, no. 1 (2024): 12–14, 10.1016/S2213-8587(23)00354-6. 38048797

[9] E. Bonifacio , C. Winkler , P. Achenbach , and A.‐G. Ziegler , “Effect of Population‐Wide Screening for Presymptomatic Early‐Stage Type 1 Diabetes on Paediatric Clinical Care,” Lancet Diabetes & Endocrinology 12, no. 6 (2024): 376–378, 10.1016/S2213-8587(24)00101-3. 38723647

[10] P. Mayring and T. Fenzl , “Qualitative Inhaltsanalyse,” in Handbuch Methoden der empirischen Sozialforschung, ed. N. Baur and J. Blasius (Springer Fachmedien Wiesbaden, 2019), 633–648.

[11] L. J. Damschroder , D. C. Aron , R. E. Keith , S. R. Kirsh , J. A. Alexander , and J. C. Lowery , “Fostering Implementation of Health Services Research Findings Into Practice: A Consolidated Framework for Advancing Implementation Science,” Implementation Science 4, no. 1 (2009): 50, 10.1186/1748-5908-4-50. 19664226 PMC2736161

[12] J. Mari , S. Solidoro , C. Braida , G. Tamaro , E. Faleschini , and G. Tornese , “Perceptions and Understanding of Family Pediatricians Regarding the New Italian Type 1 Diabetes Screening Program,” Diabetes Research and Clinical Practice 218 (2024): 111931, 10.1016/j.diabres.2024.111931. 39536974

[13] E. Ospelt , H. Hardison , N. Rioles , et al., “Understanding Providers' Readiness and Attitudes Toward Autoantibody Screening: A Mixed‐Methods Study,” Clinical Diabetes 42, no. 1 (2023): 17–26, 10.2337/cd23-0057. 38230325 PMC10788649

[14] M. Phillip , P. Achenbach , A. Addala , et al., “Consensus Guidance for Monitoring Individuals With Islet Autoantibody‐Positive Pre‐Stage 3 Type 1 Diabetes,” Diabetologia 67, no. 9 (2024): 1731–1759, 10.1007/s00125-024-06205-5. 38910151 PMC11410955

[15] J. M. Wentworth , A. B. E. Sing , G. Naselli , et al., “Islet Autoantibody Screening Throughout Australia Using in‐Home Blood Spot Sampling: 2‐Year Outcomes of Type1Screen,” Diabetes Care 48, no. 4 (2025): 556–563, 10.2337/dc24-2443. 39879258

[16] A. B. E. Sing , G. Naselli , D. Huang , et al., “Feasibility and Validity of in‐Home Self‐Collected Capillary Blood Spot Screening for Type 1 Diabetes Risk,” Diabetes Technology & Therapeutics 26, no. 2 (2024): 87–94, 10.1089/dia.2023.0345. 37976038

[17] K. M. Simmons , E. Youngkin , A. Alkanani , et al., “Screening Children for Type 1 Diabetes‐Associated Antibodies at Community Health Fairs,” Pediatric Diabetes 20, no. 7 (2019): 909–914, 10.1111/pedi.12902. 31376227 PMC6786926

[18] S. Hummel , M. Koeger , E. Bonifacio , and A. G. Ziegler , “Dysglycaemia Definitions and Progression to Clinical Type 1 Diabetes in Children With Multiple Islet Autoantibodies,” Lancet Diabetes and Endocrinology 13, no. 1 (2025): 10–12, 10.1016/s2213-8587(24)00337-1. 39642898

[19] K. Vehik , D. Boulware , M. Killian , et al., “Rising Hemoglobin A1c in the Nondiabetic Range Predicts Progression of Type 1 Diabetes as Well as Oral Glucose Tolerance Tests,” Diabetes Care 45, no. 10 (2022): 2342–2349, 10.2337/dc22-0828. 36150054 PMC9587339

[20] P. F. Teixeira , T. Battelino , A. Carlsson , et al., “Assisting the Implementation of Screening for Type 1 Diabetes by Using Artificial Intelligence on Publicly Available Data,” Diabetologia 67, no. 6 (2024): 985–994, 10.1007/s00125-024-06089-5. 38353727 PMC11058797

[21] F. M. Karl , C. Winkler , A. G. Ziegler , M. Laxy , and P. Achenbach , “Costs of Public Health Screening of Children for Presymptomatic Type 1 Diabetes in Bavaria, Germany,” Diabetes Care 45, no. 4 (2022): 837–844, 10.2337/dc21-1648. 35156126

[22] R. B. Silver , R. P. Newland , K. Hartz , et al., “Integrating Early Childhood Screening in Pediatrics: A Longitudinal Qualitative Study of Barriers and Facilitators,” Clinical Practice in Pediatric Psychology 5, no. 4 (2017): 426–440, 10.1037/cpp0000214.

[23] H. L. Rogers , S. Pablo Hernando , S. Núñez ‐ Fernández , et al., “Barriers and Facilitators in the Implementation of an Evidence‐Based Health Promotion Intervention in a Primary Care Setting: A Qualitative Study,” Journal of Health Organization and Management 35, no. 9 (2021): 349–367, 10.1108/JHOM-12-2020-0512. PMC9136863 34464035

