# Supplementary material for: Health Care Provider Perceptions of Screening for Early‐Stage Type 1 Diabetes—A Survey Study
Source: Diabetes Obes Metab. 2026 Apr 22;28(7):5939–51. doi: 10.1111/dom.70803 (PMC13243973; doi:10.1111/dom.70803)
Supplement: Supplementary file 1 — Table S1: Estimated time per child for early‐stage Type 1 diabetes screening and monitoring. Data are shown as total, reflecting responses of all responding primary healthcare providers respectively specialised paediatric diabetes centres, and stratified by the number of children screened respectively under care per month. Figure S1:. Flow‐chart of the procedures and responsibilities within the early‐stage Type 1 diabetes screening programme. Figure S2:. Feasibility of screening for early‐stage Type 1 diabetes within a research setting, as reported by primary health care providers. Figure S3:. Feasibility of monitoring children with early‐stage Type 1 diabetes within a research setting, as reported by specialised paediatric diabetes centres. Methods S1. Survey items for primary and specialised HCP. [file DOM-28-5939-s001.docx]

Supplementary Material

**Health Care Provider Perceptions of Screening for Early-stage Type 1 Diabetes – a Survey Study**

Content

| Supplementary Table 1 | Estimated time per child for early-stage type 1 diabetes screening and monitoring. | Page 2 |
| --- | --- | --- |
| Supplementary Figure S1 | Flow-chart of the procedures and responsibilities within the early-stage type 1 diabetes screening programme (Fr1da study). | Page 3 |
| Supplementary Figure S2 | Feasibility of screening for early-stage type 1 diabetes within a research setting, as reported by primary health care providers. | Page 4 |
| Supplementary Figure S3 | Feasibility of monitoring children with early-stage type 1 diabetes within a research setting, as reported by specialised paediatric diabetes centres. | Page 5 |
| Supplementary Methods 1 | Transcripts of survey items for primary and specialised HCP | Page 6 |

**Supplementary Table 1**: Estimated time per child for early-stage type 1 diabetes screening and monitoring. Data are shown as total, reflecting responses of all responding primary healthcare providers respectively specialised paediatric diabetes centres, and stratified by the number of children screened per month respectively under care.

| **Primary healthcare providers** | | | | |
| --- | --- | --- | --- | --- |
|  | **Total**  N = 194 | **< 10 Children screened/month** N = 118 | **≥ 10 Children screened/month** N = 76 | ***p*-value** |
| Time* for screening per child with negative screening result (min), Median (IQR) | 17 (13, 22) | 19 (15, 26) | 15 (12, 18) | <0.001 |
| Unknown, n | 15 | 13 | 2 |  |
| Time* for screening per child with diagnosis of early-stage T1D (min), Median (IQR) | 49 (38, 59) | 53 (42, 63) | 43 (37, 54) | 0.003 |
| Unknown, n | 51 | 41 | 10 |  |
|  |  |  |  |  |
| **Specialised paediatric diabetes centres** | | | | |
|  | **Total**  N = 10 | **< 10 Children under care** N = 4 | **≥ 10 Children under care** N = 6 | ***p*-value** |
| Time* for staging per visit (min),  Median (IQR) | 138 (125, 175) | 125 (118, 175) | 143 (135, 175) | 0.4 |
| Time* for monitoring per visit (min),  Median (IQR) | 75 (60, 85) | 75 (65, 105) | 73 (55, 85) | 0.7 |

*Time estimates were based on self-reported cumulative time needed to perform all screening respectively staging and monitoring procedures, outlined in Supplementary Figure S1; IQR = Interquartile range

Supplementary Figure S1

Supplementary Figure S1: Flow-chart of the procedures and responsibilities within the early-stage type 1 diabetes screening programme (Fr1da-study).


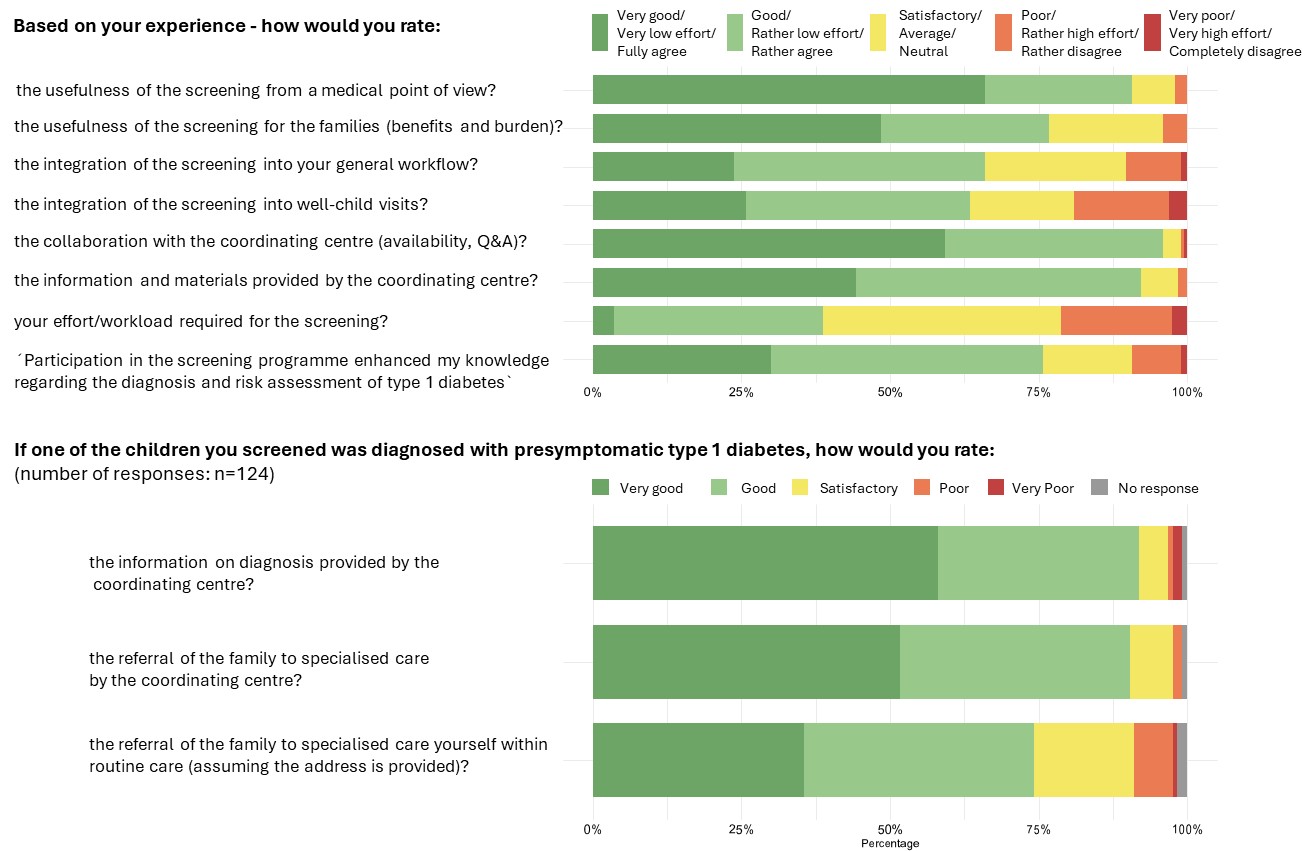


Supplementary Figure S2: Feasibility of screening for early-stage type 1 diabetes within a research setting, as reported by primary health care providers.


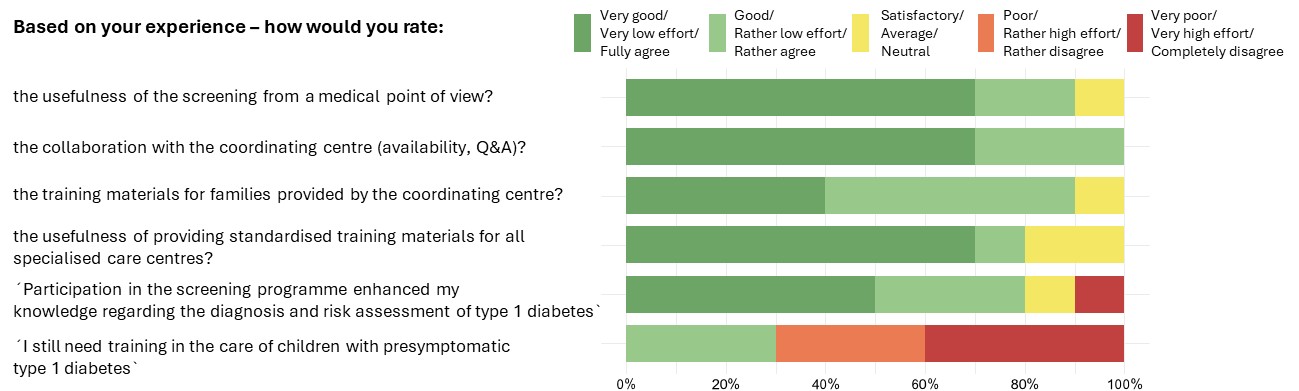


Supplementary Figure S3: Feasibility of monitoring children with early-stage type 1 diabetes within a research setting, as reported by specialised paediatric diabetes centres.

### **Supplementary Methods 1**

### **Questionnaire Transcripts**

1. **Transcript of online questionnaire for primary care paediatric practices (English translation)**
2. Who fills out this questionnaire?

☐ Physician ☐ Nurse ☐ Other: ____

1. How long has your practice been participating in the Fr1da study?

☐ Less than 1 year ☐ 1 to 5 years ☐ More than 5 years

1. On average, how many children per month participate in the Fr1da study at your practice?

Number: _____

1. What proportion of families decide not to participate despite being informed about the study?

Please provide the proportion in percent: _____ % ☐ Unknown

1. If your practice actively recruits families for participation in the Fr1da study, who usually performs this recruitment?

☐ Physician ☐ Nurse ☐ Other: ____ ☐ No active recruitment is performed

1. On average, how much time does recruitment take per child?

_____ minutes

1. **Each child can participate in the Fr1da study twice, provided that no early stage of type 1 diabetes is detected. Do you actively inform families in your practice about the possibility of repeat testing?**

☐ Yes ☐ No ☐ I was not aware of this possibility

1. **What percentage of children in your practice have undergone a second screening?**

_____ % ☐ Unknown

1. Who usually informs families about the screening and obtains informed consent from the parents?

☐ Physician ☐ Nurse ☐ Other: ____

1. On average, how much time does the information session and consent process take per child?

_____ minutes

1. Who usually performs the capillary blood draw required for participation in the Fr1da study?

☐ Physician ☐ Nurse ☐ Other: ____

1. On average, how much time does the capillary blood draw take per child?

_____ minutes

1. Who usually performs the venous blood draw when required for individual children?

☐ Physician ☐ Nurse ☐ Other: ____
☐ No venous blood draws have been performed yet as part of the Fr1da study

1. On average, how much time does the venous blood draw take per child?

_____ minutes

1. Who is usually responsible for packaging and shipping the blood samples in your practice?

☐ Physician ☐ Nurse ☐ Other: ____

1. On average, how much time does packaging and shipping the blood samples take per shipment?

_____ minutes

1. How often do you ship blood samples as part of the Fr1da study?

☐ Several times per week ☐ Once a week ☐ Every two weeks ☐ Once a month
☐ Less than once a month

1. When the Fr1da team informs you that a child has tested positive, it is your task to inform the family. Who usually conducts this conversation?

☐ Physician ☐ Nurse ☐ Other: ____ ☐ Has not yet occurred

1. On average, how much time does this conversation take per child?

_____ minutes

1. If the Fr1da team asks you to send a second (venous) blood sample, you need to contact the parents and arrange an appointment. Who usually conducts this conversation?

☐ Physician ☐ Nurse ☐ Other: ____ ☐ Has not yet occurred

1. On average, how much time does this conversation take per child?

_____ minutes

1. For children who test negative, with what percentage of families do you nonetheless have a conversation regarding the negative result?

_____ % ☐ No conversations are conducted

1. Who usually conducts this conversation?

☐ Physician ☐ Nurse ☐ Other: ____

1. On average, how much time does this conversation take per child?

_____ minutes

1. **Based on your experience of the Fr1da study in recent years, ...**

How useful do you think the screening is from a medical point of view?

□ Very useful □ Somewhat useful □ Average □ Not particularly useful □ Not at all useful

How useful do you think the screening is for the families (including the opportunities and burden involved)?

□ Very useful □ Somewhat useful □ Average □ Not particularly useful □ Not at all useful

How can the screening be integrated into the general workflow in your practice?

□ Very good □ Good □ Satisfactory □ Poor □ Very poor

How can the screening be integrated into the routine paediatric well-child-visits in particular?

□ Very good □ Good □ Satisfactory □ Poor □ Very poor

How would you rate the effort required for the screening in your practice?

□ Very low □ Rather low □ Neutral □ Rather high □ Very high

How would you rate the information and materials you have received from the coordinating centre for the Fr1da study?

□ Very good □ Good □ Satisfactory □ Poor □ Very poor

How would you rate the collaboration with the coordinating centre (availability, responses provided, etc.)?

□ Very good □ Good □ Satisfactory □ Poor □ Very poor

"Participation in the Fr1da study enhanced my knowledge regarding the diagnosis and risk assessment of type 1 diabetes."

□ Fully agree □ Rather agree □ Neutral □ Rather disagree □ Completely disagree

1. **Regarding the integration of screening into routine care...**

How feasible do you consider integrating the screening into paediatric routine preventive care in general?

□ Very good □ Good □ Satisfactory □ Poor □ Very poor

How feasible do you consider each of the individual screening components?

Educating families about the screening

□ Very good □ Good □ Satisfactory □ Poor □ Very poor

Blood draws

□ Very good □ Good □ Satisfactory □ Poor □ Very poor

Packaging and shipping of blood samples

□ Very good □ Good □ Satisfactory □ Poor □ Very poor

Communicating positive test results to families

□ Very good □ Good □ Satisfactory □ Poor □ Very poor

Would a screening method using a dried blood filter card (similar to newborn screening) be more feasible for you than the current capillary blood sampling?

□ Yes □ No □ I don’t know

What further challenges do you see in integrating the screening into routine care?

___________________________________________________________________________

1. **Assuming that the screening is initially implemented only for first-degree relatives of people with type 1 diabetes...**

How feasible would you consider implementing screening for first-degree relatives only, compared to universal screening?

□ Much better □ Slightly better □ Comparable □ Slightly worse □ Much worse

How feasible do you consider assessing family history of type 1 diabetes beforehand?

□ Very good □ Good □ Satisfactory □ Poor □ Very poor

1. **Have one or more children in your practice been diagnosed with an early stage of type 1 diabetes ((detection of 2 or more diabetes-specific autoantibodies)?**

□ Yes □ No

If yes, how would you rate...

the information on diagnosis provided by the coordinating centre?

□ Very good □ Good □ Average □ Poor □ Very poor

the referral of the family to a paediatric diabetes care centre by the coordinating centre?

□ Very good □ Good □ Average □ Poor □ Very poor

referring the family to a diabetes centre yourself within routine care (assuming you receive the centre's contact details)

□ Very good □ Good □ Average □ Poor □ Very poor

1. **As part of monitoring children with early-stage type 1 diabetes, regular follow-up examinations (HbA1c and blood glucose measurements) are required. Are you already performing such tests in your practice within the Fr1da study?**

□ Yes □ No □ I don’t know

Regardless of whether you currently conduct monitoring, how feasible would you find intermittent follow-up testing (HbA1c/glucose) in your practice?

□ Very good □ Good □ Satisfactory □ Poor □ Very poor

Which aspects specifically limit the feasibility of follow-up testing in your practice? (Multiple answers possible)

☐ Blood draws
☐ Sample shipping and analysis in the laboratory
☐ Interpretation of results and communication of results with families
☐ Coordination of follow-up appointments
☐ Referring families to a diabetes centre in case of abnormal results
☐ Other: ____

1. **Are there any other concerns, costs, or resource requirements related to participation in the Fr1da study that have not yet been addressed?**

___________________________________________________________________________

1. **Transcript of online** **questionnaire for paediatric diabetes care centres (English translation)**
2. Who fills out this questionnaire?

☐ Physician ☐ Nurse ☐ Diabetes advisor ☐ Other: ____

1. How long has your diabetes care centre been participating in the Fr1da study?

☐ Less than 1 year ☐ 1 to 5 years ☐ More than 5 years

1. How many children are currently under care in your centre as part of the Fr1da study?

Number: _____

1. After the diagnosis of early-stage type 1 diabetes, families are informed about it by the Fr1da coordinating centre. Who usually arranges the appointment for the initial appointment for training and metabolic staging (HbA1c and oral glucose tolerance test (OGTT)) at your centre?

☐ Physician ☐ Nurse ☐ Diabetes advisor ☐ Other: ____

1. On average, how much time does scheduling this appointment take per child?

_____ minutes

1. Who usually conducts the initial education and training session for families with a child diagnosed with early-stage type 1 diabetes?

☐ Physician ☐ Nurse ☐ Diabetes advisor ☐ Other: ____

1. On average, how much time does the initial education and training session take per child?

_____ minutes

1. When is the initial education and training session conducted?

☐ During waiting-times of the OGTT ☐ Before or after the OGTT (same day)

☐ On a separate appointment then the OGGT ☐ Other: ____

1. Who usually performs the children’s venous blood draws for blood glucose and HbA1c measurement?

☐ Physician ☐ Nurse ☐ Diabetes advisor ☐ Other: ____

1. On average, how much time does the venous blood draw take per child?

_____ minutes

1. Who usually prepares the OGTTs (such as the preparation of the weight-adapted glucose solution)?

☐ Physician ☐ Nurse ☐ Diabetes advisor ☐ Other: ____

1. On average, how much time does the preparation of the OGTT take per child?

_____ minutes

1. Who usually performs the children’s venous blood draws for OGTTs (or places the venous catheter)?

☐ Physician ☐ Nurse ☐ Diabetes advisor ☐ Other: ____

1. On average, how much time do the blood draws for the OGTT take in total per child (sum for all 5 time points: 0, 30, 60, 90, and 120 minutes)?

_____ minutes

1. Where is the analysis of the venous blood samples (OGTT, HbA1c) usually performed?

☐ In-house laboratory ☐ External laboratory ☐ Other: ____

1. Who is usually responsible for packaging and shipping the blood samples in your centre?

☐ Physician ☐ Nurse ☐ Diabetes advisor ☐ Other: ____

1. On average, how much time does packaging and shipping the blood samples take per shipment?

_____ minutes

1. Do you conduct conversations with families regarding the results of metabolic staging, in addition to the Fr1da coordinating centre?

☐ Yes, always ☐ Yes, as required ☐ No ☐ I don’t know

1. What proportion of families do you usually have a conversation with regarding the results of metabolic staging?

______ % ☐ I don’t know

1. Who usually conducts this conversation?

☐ Physician ☐ Nurse ☐ Diabetes advisor ☐ Other: ____

1. On average, how much time does this conversation take per child?

_____ minutes

1. Are there any other time or material expenses related to participation in the Fr1da study that have not yet been covered?

___________________________________________________________________________

1. **Based on previous experience of caring for children with an early stage of type 1 diabetes in recent years, ...**

How useful do you think the screening for islet autoantibodies is from a medical point of view?

□ Very useful □ Somewhat useful □ Average □ Not particularly useful □ Not at all useful

How would you rate the collaboration with the coordinating centre (care for families, responses and information provided, availability)

□ Very good □ Good □ Average □ Poor □ Very poor

"Participation in the Fr1da study enhanced my knowledge\n regarding the diagnosis and risk assessment of type 1 diabetes."

□ Fully agree □ Rather agree □ Neutral □ Rather disagree □ Completely disagree

"I still have a need for training in the care of children with early-stage type 1 diabetes."

□ Fully agree □ Rather agree □ Neutral □ Rather disagree □ Completely disagree

1. **Compared to families who have not participated in a screening program:**

How would you assess the following aspects for families you care for in the context of the Fr1da study?

The amount of work required for your centre to provide the initial training

□ Significantly less □ Somewhat less □ Similar □ Somewhat more □ Much more

The burden on families at the time of initiating insulin therapy

□ Significantly less □ Somewhat less □ Similar □ Somewhat greater □ Much greater

The amount of work required for your centre to provide the initial insulin treatment

□ Significantly less □ Somewhat less □ Similar □ Somewhat more □ Much more

The proportion of children who could be treated on an outpatient basis at the start of insulin therapy (Assuming reimbursement is possible with the health insurance provider)

□ Significantly less □ Somewhat less □ Similar □ Somewhat more □ Much more

The amount of work required for your centre to provide long-term care

□ Significantly less □ Somewhat less □ Similar □ Somewhat more □ Much more

1. **What advantages or disadvantages do you see for your paediatric diabetes care centre and for the families through the screening program?**

**___________________________________________________________________________**

1. **Which materials do you currently use in your centre for educating and training families? (Multiple answers possible)**

☐ Own training materials
☐ Training materials provided by the Fr1da coordinating centre (Fr1da brochure, presentation slides)
☐ Other: ____________________
☐ No specific materials are used for education

1. **How would you rate the training materials provided by the Fr1da coordinating centre for the families?**

□ Very good □ Good □ Average □ Poor □ Very poor

1. **How useful do you find the provision of standardized training materials for all diabetes care centres?**

□ Very useful □ Somewhat useful □ Average □ Not particularly useful □ Not at all useful

1. **Are there any materials or topics that you think should be added to the current educational materials?**

___________________________________________________________________________

1. **How would you rate the feasibility of the following procedures in the context of the Fr1da study for your centre?**

OGTT

□ Very good □ Good □ Satisfactory □ Poor □ Very poor □ No answer

HbA1c measurement (in the laboratory)

□ Very good □ Good □ Satisfactory □ Poor □ Very poor □ No answer

Venous blood glucose measurement (in the laboratory)

□ Very good □ Good □ Satisfactory □ Poor □ Very poor □ No answer

Capillary blood glucose measurement with a quality-controlled glucose meter on site

□ Very good □ Good □ Satisfactory □ Poor □ Very poor □ No answer

1. **How would you rate the feasibility of the following additional follow-up examinations in the context of Fr1da for your education centre?**

Training families in blood glucose self-monitoring at home and assessing the measurement results

□ Very good □ Good □ Satisfactory □ Poor □ Very poor □ No answer

Sensor-based / continuous glucose monitoring (CGM)

□ Very good □ Good □ Satisfactory □ Poor □ Very poor □ No answer

1. **Which of the following follow-up examinations do you consider the most challenging, and why?**

(Multiple answers possible)

☐ Oral glucose tolerance test (OGTT): ____
☐ HbA1c measurement in the laboratory: ____
☐ Venous blood glucose measurement in the laboratory: ____
☐ Capillary blood glucose measurement with a quality-controlled glucose meter on site: ____
☐ Blood glucose self-monitoring by the family at home: ____
☐ Sensor-based / continuous glucose monitoring (CGM): ____
☐ None

1. **Which of the following follow-up examinations do you prefer, and why?**

(Multiple answers possible)

☐ Oral glucose tolerance test (OGTT): ____
☐ HbA1c measurement in the laboratory: ____
☐ Venous blood glucose measurement in the laboratory: ____
☐ Capillary blood glucose measurement with a quality-controlled glucose meter on site: ____
☐ Blood glucose self-monitoring by the family at home: ____
☐ Sensor-based / continuous glucose monitoring (CGM): ____
☐ None

1. **Which of the following would be a more feasible alternative to the standard OGTT (venous blood draws at 5 time points: 0, 30, 60, 90, and 120 minutes)?**

(Multiple answers possible)

☐ Performing the OGTT exclusively using capillary blood glucose measurements
☐ Reducing the number of time points
☐ Replacing the OGTT with continuous glucose monitoring (CGM)
☐ Other: ____

1. **How would you rate the acceptance of the following follow-up examinations by families in the context of the Fr1da study?**

OGTT

□ Very good □ Good □ Satisfactory □ Poor □ Very poor □ No answer

HbA1c measurement (in the laboratory)

□ Very good □ Good □ Satisfactory □ Poor □ Very poor □ No answer

Venous blood glucose measurement (in the laboratory)

□ Very good □ Good □ Satisfactory □ Poor □ Very poor □ No answer

Capillary blood glucose measurement with a quality-controlled glucose meter on site

□ Very good □ Good □ Satisfactory □ Poor □ Very poor □ No answer

Blood glucose self-monitoring by families at home

□ Very good □ Good □ Satisfactory □ Poor □ Very poor □ No answer

Sensor-based / continuous glucose monitoring (CGM)

□ Very good □ Good □ Satisfactory □ Poor □ Very poor □ No answer

1. **Are there any measures that could make care for families in the diabetes care centres easier or more pleasant?**

___________________________________________________________________________

1. **Assuming that islet autoantibody screening is integrated into routine medical care…**

How feasible do you consider staging and follow-up of children with early-stage type 1 diabetes within routine care?

□ Very good □ Good □ Satisfactory □ Poor □ Very poor

How useful do you find offering family education and training sessions in the form of online group sessions?

□ Very useful □ Somewhat useful □ Average □ Not particularly useful □ Not at all useful

1. **How feasible do you consider it for your centre to take over the following tasks, currently managed by the Fr1da team, within routine care?**

Initial consultation and education of families about the diagnosis and further procedures

□ Very good □ Good □ Satisfactory □ Poor □ Very poor

Procurement and provision of testing materials for staging and follow-up (e.g., OGTT solutions)

□ Very good □ Good □ Satisfactory □ Poor □ Very poor

Procurement and provision of materials for home blood glucose monitoring (device, test strips, lancets)

□ Very good □ Good □ Satisfactory □ Poor □ Very poor

Interpretation of laboratory results and communicating findings to the family

□ Very good □ Good □ Satisfactory □ Poor □ Very poor

Creation of individualized follow-up plans

□ Very good □ Good □ Satisfactory □ Poor □ Very poor

Coordination of follow-up appointments for follow-up examinations

□ Very good □ Good □ Satisfactory □ Poor □ Very poor

1. **Which of the above-mentioned tasks do you consider the greatest challenge, and what other challenges do you see regarding integration into routine care?**

___________________________________________________________________________

1. **Do you have any other feedback or suggestions you would like to share with us?**

________________________________________________________________________________
